# Revisiting Fishery Sustainability Targets

**DOI:** 10.1007/s11538-024-01352-7

**Published:** 2024-09-16

**Authors:** Vincent Cattoni, Leah F. South, David J. Warne, Carl Boettiger, Bhavya Thakran, Matthew H. Holden

**Affiliations:** 1 The University of Queensland School of Mathematics and Physics, Saint Lucia, Australia; 2 School of Mathematical Sciences, Queensland University of Technology, Brisbane, Australia; 3 Present Address: Centre for Data Science, Queensland University of Technology, Brisbane, Australia; 4 Department of Environmental Science, Policy and Management, University of California, Berkeley, Berkeley USA; 5 Centre for Biodiversity and Conservation Science, The University of Queensland, Saint Lucia, Australia; 6 Centre for Marine Science, The University of Queensland, Saint Lucia, Australia

**Keywords:** Optimal escapement, Maximum sustainable yield, Fisheries management, Bayesian model selection, Fishery reference points, Harvest strategies

## Abstract

Density-dependent population dynamic models strongly influence many of the world’s most important harvest policies. Nearly all classic models (e.g. Beverton-Holt and Ricker) recommend that managers maintain a population size of roughly 40–50 percent of carrying capacity to maximize sustainable harvest, no matter the species’ population growth rate. Such insights are the foundational logic behind most sustainability targets and biomass reference points for fisheries. However, a simple, less-commonly used model, called the Hockey-Stick model, yields very different recommendations. We show that the optimal population size to maintain in this model, as a proportion of carrying capacity, is one over the population growth rate. This leads to more conservative optimal harvest policies for slow-growing species, compared to other models, if all models use the same growth rate and carrying capacity values. However, parameters typically are not fixed; they are estimated after model-fitting. If the Hockey-Stick model leads to lower estimates of carrying capacity than other models, then the Hockey-Stick policy could yield lower absolute population size targets in practice. Therefore, to better understand the population size targets that may be recommended across real fisheries, we fit the Hockey-Stick, Ricker and Beverton-Holt models to population time series data across 284 fished species from the RAM Stock Assessment database. We found that the Hockey-Stick model usually recommended fisheries maintain population sizes higher than all other models (in 69–81% of the data sets). Furthermore, in 77% of the datasets, the Hockey-Stick model recommended an optimal population target even higher than 60% of carrying capacity (a widely used target, thought to be conservative). However, there was considerable uncertainty in the model fitting. While Beverton-Holt fit several of the data sets best, Hockey-Stick also frequently fit similarly well. In general, the best-fitting model rarely had overwhelming support (a model probability of greater than 95% was achieved in less than five percent of the datasets). A computational experiment, where time series data were simulated from all three models, revealed that Beverton-Holt often fit best even when it was not the true model, suggesting that fisheries data are likely too small and too noisy to resolve uncertainties in the functional forms of density-dependent growth. Therefore, sustainability targets may warrant revisiting, especially for slow-growing species.

## Introduction

Natural resource managers and scientists have largely regarded harvesting maximum sustainable yield as problematic for conservation (Pauly and Froese 2021). This is because such decisions risk population collapse and even extinction when applied to systems that suffer from uncertainty, random fluctuations, or low initial population sizes (Clark 2010; Wright 1983; Kirkwood 1981). Therefore the primary goal of modern harvest management is to maintain a healthy-sized population that replenishes itself. Framing harvest policies based on, “what population size should we maintain?" rather than “how much can we extract?" allows us, in theory, to buffer against population collapse in the presence of unpredictability. It has been shown across a wide range of population dynamic models that to optimize sustainable yield in random environments, it is better to set a population size target, and only extract surplus individuals above the target than to set a catch target. This logic underpins much of the world’s sustainability goals for fisheries and other harvested populations (Earle 2021; Kemp et al. 2020).

But what target population size should we aim to maintain? In a classic paper, Reed (1979) showed that for a large set of simple population dynamic models and types of random fluctuations, letting a fixed population size “escape” harvest maximizes long-run sustainable harvest. The specific optimal escapement rule is the population size that maximizes the productivity of the stock. Surprisingly, when solving this condition, nearly all models lead to levels near 50 percent of carrying capacity or lower (Holden and Ellner 2016). This is regardless of the ecological parameters governing population growth. In other words, slow-growing species should have the same escapement targets as fast-growing species in these models, as a proportion of carrying capacity. While modifications of this work can suggest minor changes in the optimal escapement policy (Sethi et al. 2005), the recommendation of maintaining populations at or below 50 percent of carrying capacity is surprisingly robust to assumptions around stochastic fluctuations (Kirkwood 1987), uncertainty (Sethi et al. 2005), age-structure (Holden and Conrad 2015) and the details of population growth (Kapaun and Quaas 2013). Many of said modifications even lead to counter-intuitively more aggressive harvest policies (Kirkwood 1987; Sethi et al. 2005; Holden and Conrad 2015; Kapaun and Quaas 2013).

As a result, many of the world’s fisheries management agencies attempt to maintain stock sizes at or around 50–60 percent of carrying capacity (Earle 2021; Kemp et al. 2020). For example, as a precautionary approach, Australian fish stocks have a target of maintaining 60 percent of the carrying capacity in the ocean (Hutton et al. 2019; Helidoniotis 2021; Dichmont et al. 2021). This policy, or reference point, is often referred to as B60, standing for maintaining 60 percent of unfished biomass. Such targets are widely justified by the classic theory above. But are there any population dynamic models that would yield more conservative optimal escapement targets? If so, do they fit harvested population time series data better or worse than the classic models commonly used by fisheries managers and ecological theorists? These are the questions we aim to answer in this work.

## Classic Optimal Sustainability Targets

In the seminal work by Reed (1979), he started with a model of the form,

1 $$\begin{aligned} x_{t+1} = Z_t f(x_t-h_t), \end{aligned}$$

where $x_t$ is population size at time *t*, $Z_t$ is an independent identically distributed random variable with mean one, $h_t$ is the harvest amount, and *f* is some density-dependent growth function. His goal was to solve for the harvest policy that maximized the long-run sum of discounted harvest, with discount factor $\rho$. He proved that under this formulation, the optimal harvest policy is to maintain a population size of $^*$ and harvest individuals over this size threshold, where $*$ is given by the solution to

2 $$\begin{aligned} f^\prime (s^*) = \frac{1}{\rho }. \end{aligned}$$

If the population happens to be below $s^*$, it is optimal not to harvest until the population grows above this size. Because we want to produce the most conservative harvest rules possible under the classic models, we will let $\rho =1$, which corresponds to no discounting. Note that Reed actually derived a more general rule for maximizing nonlinear utility functions of harvest. However, we will examine policies that maximize the direct sum of the long-run harvest to simplify the presentation.

In this paper, we will consider a variety of commonly used growth functions *f*. To make comparisons, we will parameterize all functions in terms of a growth parameter *r* and carrying capacity *k*. We will define *r* to be equal to $f^\prime (0)$, which means it is the growth multiplier, or proliferation rate (Filar and Streipert 2022), which can be thought of as one plus the natural log of the fundamental population growth rate in continuous time models, such as the logistic differential equation. We define *k* to be the non-zero equilibrium of the model, and we will only consider models with one such equilibrium, as is standard in natural resource management.

Perhaps the most widely used model in fisheries is the Beverton-Holt model (Beverton and Holt 2012), which is of the form

$$\begin{aligned} f_B(x) = \frac{rx}{1+(r-1)x/k}. \end{aligned}$$

It is a convenient model because it always leads to stable population dynamics that monotonically approach equilibrium and the model can be mechanistically derived from the principles of contest competition (Duncan et al. 2009; Anazawa 2012; McGowan et al. 2018). By Eq. (2) optimal escapement can be solved analytically as,

3 $$\begin{aligned} s^*_B = \left( \frac{\sqrt{r} - 1}{r - 1}\right) k. \end{aligned}$$

Perhaps the second most widely used model in fisheries is the Ricker model (Ricker 1954),

$$\begin{aligned} f_R(x) = xr^{1-\frac{x}{k}}, \end{aligned}$$

which is often used to model species that experience cannibalism or other mechanisms of over-compensatory density dependence, such as scramble competition (Franco et al. 2018; Anazawa 2019; Ahrens et al. 2020). Note that the parameterization here may be less familiar than the one for which the model contains Euler’s mathematical constant *e*. The above version is mathematically equivalent to the more commonly written version, however, this version has the advantage that it is written in terms of its growth multiplier $r=f^\prime _R(0)$. This function does not yield an analytical solution for optimal escapement, however, it can be computed numerically.

## The Hockey-Stick Model and its Sustainability Target

A much less common model, especially among mathematicians, economists, and stock assessment scientists is the Hockey-Stick model (Butterworth 1993). It is a piecewise-linear model, producing exponential population growth, until abruptly capping the population size at carrying capacity, *k*. It is given by the equation,

$$\begin{aligned} f_H(x) = \text { min}(rx, k). \end{aligned}$$

**Fig. 1:**
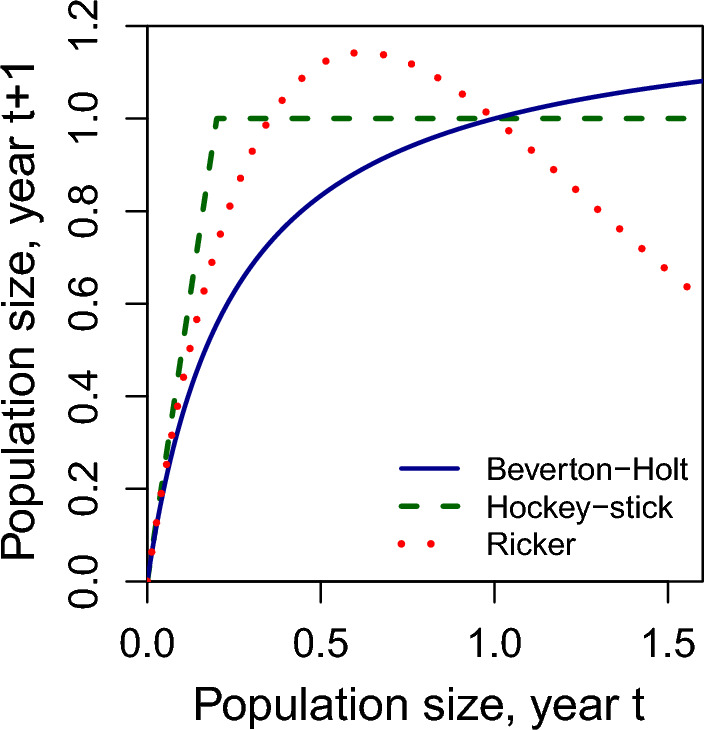
Population dynamic model curves (*r*=1.7). Population size is in units proportion of carrying capacity

Its graph has a sharp corner at $x=k/r$ and therefore, violates the differentiability assumptions of much of the previous theory. Even so, it is straightforward to show that in the absence of discounting, it is optimal to let

4 $$\begin{aligned} s^*_H = \left( \frac{1}{r}\right) k, \end{aligned}$$

escape harvest in this model (see the Appendix for a derivation). This result is due to a complete lack of density dependence for populations below size $s^*_H$. In Fig. 1, we display the Hockey-Stick model, along with the Beverton-Holt and Ricker models for a growth multiplier of $r=1.7$.

**Fig. 2:**
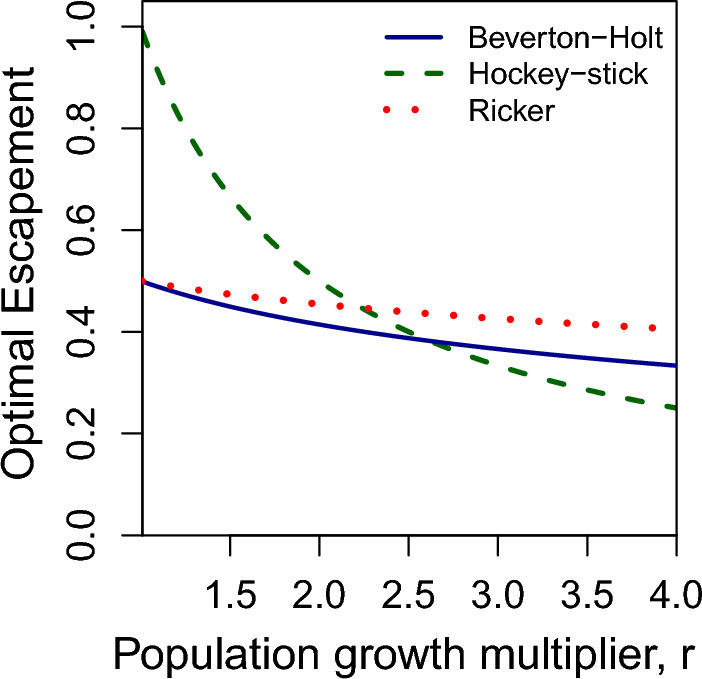
Optimal escsapement for each model as a function of the population growth rate *r*

One aspect of the models that becomes immediately clear, is that while the equations for Beverton-Holt and Ricker appear considerably different, when parameterized with the same *r* and *k* values, their graphs look remarkably similar for values of population size below carrying capacity. They only deviate considerably for population sizes above *k*. Given the similarity between the Ricker and Beverton-Holt population curves, perhaps it would not be surprising if they produced similar values for optimal escapement. Indeed, they do. In Fig. 2, which plots optimal escapement for each model as a function of the population growth multiplier, *r*, we can see that *r* has little effect on optimal escapement in the Beverton-Holt and Ricker models, which produce a target escapement of just below half of carrying capacity for all *r*. For *r* less than $\frac{3+ \sqrt{5}}{2}$, Hockey-Stick suggests a more conservative optimal escapement than Beverton-Holt. Additionally, for *r* less 5/3, the Hockey-Stick model produces optimal escapements above 60 percent of carrying capacity.

**Fig. 3:**
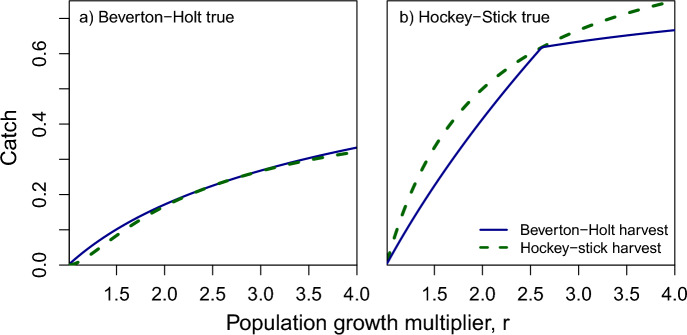
Catch when using optimal escapement rules from Beverton-Holt (solid line) and Hockey-Stick (dashed line) models, when population dynamics are governed by **a** the Beverton-Holt model and **b** the Hockey-Stick model

**Fig. 4:**
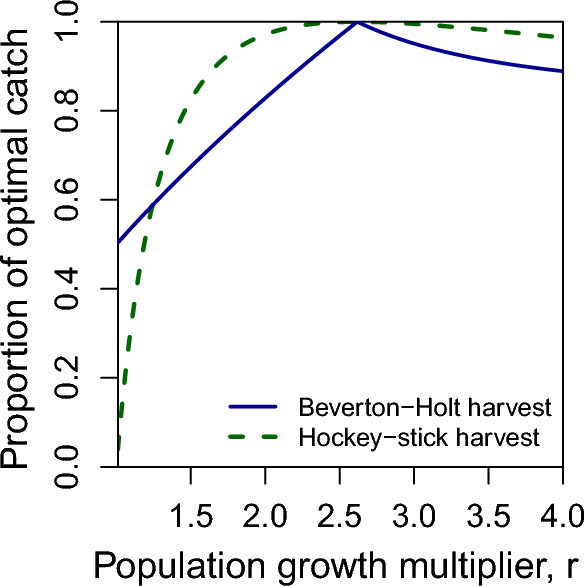
Proportion of the optimal catch achieved when using the wrong model to determine an escapement rule. The dashed curve is the proportion of optimal catch when using the Hockey-Stick escapement rule but dynamics are Beverton-Holt, and the solid curve is the proportion of optimal catch when using the Beverton-Holt escapement rule but dynamics are Hockey-Stick

What happens when you use the optimal escapement policy from one model, but the real world is governed by a different model? In Fig. 3 we see that if the real world is governed by the Beverton-Holt model (Fig. 3a), total catch is lower than if it is governed by the Hockey-Stick model (Fig. 3b), due to density dependence reducing growth at intermediate population sizes. Incorrectly using the Hockey-Stick model’s optimal escapement rule, 1/*r*, when the population grows according to the Beverton-Holt model, does not reduce catch as much as the case where the manager uses the Beverton-Holt model’s escapement rule when the population dynamics are governed by the Hockey-Stick model. In Fig. 4, we see the proportion of optimal catch achieved when using the wrong model (dashed curve, Hockey-Stick escapement rule but dynamics are Beverton-Holt) and (solid curve, Beverton-Holt escapement rule but dynamics are Hockey-Stick). For a large portion of parameter space, the Hockey-Stick escapement rule achieves near-optimal catch even when the dynamics are Beverton-Holt. However, the Beverton-Holt escapement rule in Eq. (3) performs worse when it is the incorrect model.

## Confronting Optimal Escapement with Time Series Data

Previous work has shown that the Hockey-Stick model can fit fisheries data as well as other models (Barrowman and Myers 2000), despite the sharp corner causing some challenges for fitting algorithms (Mesnil and Rochet 2010). While the fits can explain as much variation in the data as other models, the fitted parameter values can vary substantially. Barrowman and Myers (2000) found that given the same data set, the Beverton-Holt model estimates a larger population growth rate and carrying capacity than the Hockey-Stick model. Because optimal escapement in the Hockey-Stick model is *k*/*r*, lower estimates for both *r* and *k* could cancel, or if one increases more than the other it may affect optimal escapement estimation either up or down. So in this section, we set out to determine if (1) the Hockey-Stick model fits global fishing data as well as the classic population models and (2) when accounting for parameter estimation, whether optimal escapement calculations in the fitted Hockey-Stick models would be more conservative than for Beverton-Holt and other classic models.

So when confronted with real data, which model suggests a more conservative optimal escapement, Beverton-Holt, Ricker, or Hockey-Stick? To determine this we fit all three models to the population time series in the RAM Legacy Stock Assessment database, a global database of thousands of fishery stock assessments, each corresponding to a different species or location. Of the data in the database, 476 fisheries had both Biomass and Catch time series. However, some of this data is clearly modeled rather than from actual observations. This can be seen by plotting biomass in year $t+1$ against escapement in year *t*, which often leads to near smooth, deterministic, curves. We visually identified these data sets, as well as data sets with negative values for escapement, as is standard when fitting models to RAM stock assessment data (Britten et al. 2017; Hilborn et al. 2020). Removing the problematic data sets left 284 sets of non-deterministic, positive data.

To estimate the parameters and quantify model uncertainty, we used a statistical model, which is an extension of the population model in (1), described probabilistically by,

$$\begin{aligned} x_{t+1}^{(i)} \sim \text {LogNormal}(\log f_{(m)}(x_t^{(i)} - h_t^{(i)}), \sigma ^2) \text{ for } t = 1,\ldots ,T_{i}-1, \end{aligned}$$

where $f_{(m)}$ is the function $f_B$, $f_R$ or $f_H$, depending on the model (*m*) in question. The superscript (*i*) denotes the dataset of interest for $i=1,\ldots ,284$, and $T_i$ is the length of the time series in dataset *i*. It is important to note that the model assumes that error is process noise rather than observation noise. This assumption aligns perfectly with the optimisation theory in Reed (1979), which is used to derive the optimal escapements in this paper.

To perform parameter estimation and model selection we applied and compared both a Bayesian and likelihood-based approach. For the Bayesian approach, we assumed the following prior distributions on the parameters,

$$\begin{aligned} r&\sim \text {Uniform}[1,10],\\ k&\sim \text {Uniform}\left[ 0.1 \max _{t}(x_t), \,\, 10 \max _{t}(x_t)\right] ,\\ \sigma&\sim \text {HalfCauchy}(0, 1). \end{aligned}$$

For model (*m*) and dataset *i*, we estimated the posterior distribution

$$\begin{aligned} p_{(m)}(r,k,\sigma |x^{(i)},h^{(i)}) \propto p_{(m)}(x^{(i)}|r,k,\sigma ,h^{(i)})p(r,k,\sigma ), \end{aligned}$$

using the RStan package (Stan Development Team 2023). The normalising constants of the posteriors were estimated using the bridgesampling package (Gronau et al. 2020), and these estimates were then converted into a probability that each model was the true model for the given dataset. These probabilities use the prior belief that all three models are considered equally likely.

Due to the challenge of choosing uninformative priors for Bayesian inference and the fact that prior distributions on the parameters influence model selection, we also used a likelihood approach. To estimate the $(r,k,\sigma )$ parameter combination that maximized the likelihood for each model-dataset pair, we used the dfoptim package (Varadhan et al. 2023), with an initial guess set to the parameter combination with the highest evaluated likelihood in the Markov chain from the Bayesian approach. We computed the Akaike weight (Anderson and Burnham 2004) for each model to measure the relative evidence supporting each model for each data set. Further details on implementation are available in the appendix.

To validate our fitting approach for the real-world fisheries data, we also conducted a computational experiment where we fit simulated data. The goal was to determine if Bayesian and likelihood-based inference could correctly identify a true model, the one used to simulate the data, out of the set of all three candidate models. To do this, we simulated twenty datasets per model, each of which required a sensible choice of *r*, *k*, $\sigma$, $x_0$, and the harvests. To select these values, we randomly selected an observed dataset, out of the 284 possible datasets, and used the corresponding observed $x_0$. We chose the values of *r*, *k*, and $\sigma$ by selecting the final value of the Markov chain for each of the specified model-dataset pairs because this (approximately) represents a draw from the posterior. We then simulated the time series data using formula (1), with the chosen parameter values. To specify the harvests in (1), we could not use the observed harvests from the selected dataset because this could lead to negative biomass given the new simulated noise. Instead, we simulated harvests by drawing, with replacement, observed harvest proportions, which we converted to absolute harvest by multiplying the drawn proportion by the simulated biomass value. The process was repeated 20 times to give 20 simulated datasets per model.

## Results from Fitting Data in RAM Stock Assessment Database

**Fig. 5:**
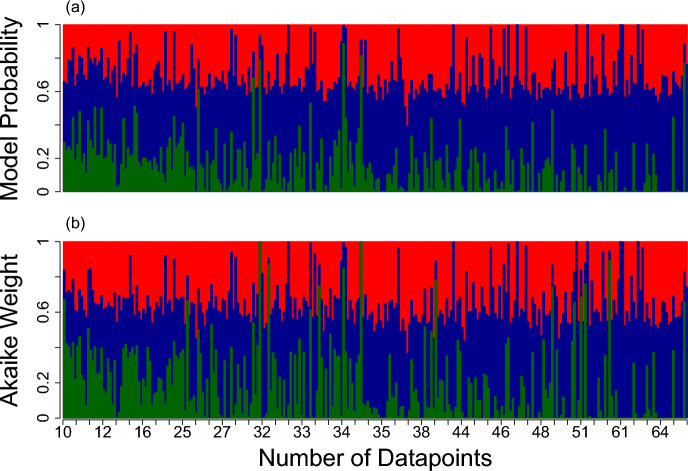
Model probabilities from our Bayesian analysis, across all 284 fisheries biomass time-series data-sets in RAM legacy Stock Assessment Database, sorted by the number of data points in each time series. Each bar sums to one, with the green portion denoting the relative support for the Hockey-Stick model by the time series data. Blue and red bars denote support for the Beverton-Holt and Ricker models respectively (Color figure online)

Using the Bayesian inference approach, Hockey-Stick, Beverton-Holt, and Ricker had the highest model probabilities in 9%, 90%, and 1% of the 284 data sets, respectively (Fig. 5). While Beverton-Holt (blue middle bars in Fig. 5a) was the most supported model in the majority of the data sets, it is important to note that the other models also received substantial support, with probabilities typically exceeding 20% even when not considered the most supported model.

Under the likelihood approach, support shifted away from Beverton-Holt when compared to the Bayesian approach, with Hockey-Stick, Beverton-Holt, and Ricker having the highest Akaike weights in 24%, 66%, and 10% of the data sets, respectively (Fig. 5b).

**Fig. 6:**
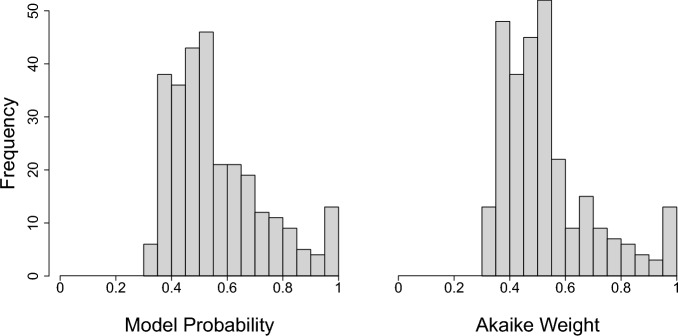
Histogram of the model probability and Akaike weight of the best fitting model for each of the 284 data-sets in RAM legacy Stock Assessment Database

In most of the 284 data sets, a substantial proportion of evidence supports each of the three models, regardless of the methodology used to perform model selection (Fig. 5). Only 5 percent had a single model achieve a model probability or Akaike weight of 95 percent or higher. The median model probability and Akaike weight of the best model across the data sets was 0.52 and 0.50, respectively (Fig. 6). Note that 1/3 or (0.33) represents all models being equally likely. So, the typical support for the best model was quite low, considering it was rarely above twice its theoretical minimum.

Of the 13 data sets that had substantial support from one model (greater than 95 percent Akaike weight), 2 supported the Hockey-Stick Model and 11 supported the Beverton-Holt model. Under the Bayesian model probability approach, there were 13 data sets with the most supported model achieving a model probability greater than 95 percent.

**Fig. 7:**
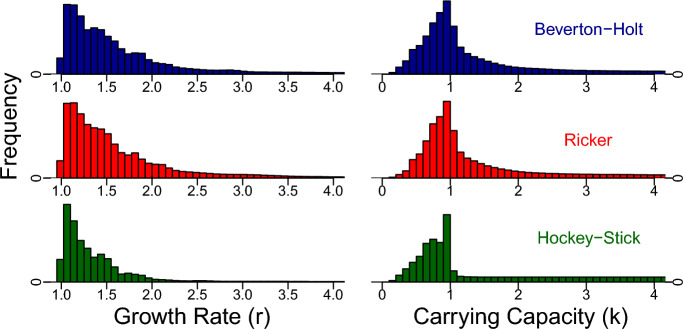
Fitted growth rate, *r*, left column and carrying capacity *k* (as a proportion of max observed biomass) combined across all posterior distributions for each dataset in the Bayesian approach. Hockey-Stick often leads to lower estimates of both growth rate and carrying capacity (more area to the left in the bottom row)

Confirming the results of Barrowman and Myers (2000), we found that the Hockey-Stick model estimates *r* to be lower (median of 1.27 across all posteriors in the Bayesian approach and 1.29 across the 284 maximum likelihood estimates) than estimates from the Beverton-Holt (1.49 and 1.57) and Ricker (1.43 and 1.51) models (Fig. 7).

**Fig. 8:**
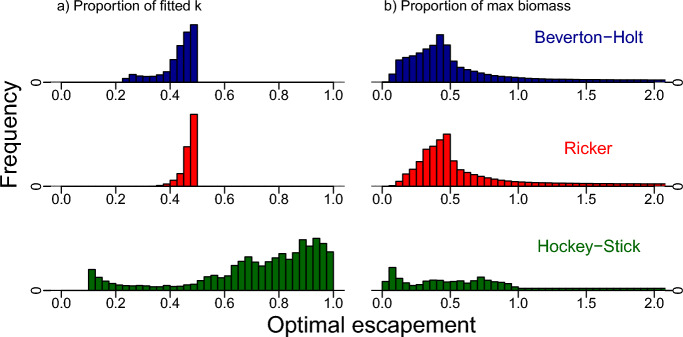
Histograms of optimal escapement for each model across all Markov-chain runs and all data sets **a** as a proportion of the fitted carrying capacity and **b** as a proportion of the maximum observed population size for the data sets in the RAM Stock Assessment Database. In **a** we see the Hockey-Stick model frequently leads to optimal escapements close to carrying capacity (green bars on the right), whereas the other models produce optimal escapements at or below 50 percent of carrying capacity. Similarly in **b** the Hockey-Stick model more often than not leads to higher total optimal escapements than the other models, but not always, due to lower fitted Hockey-Stick carrying capacities. Distributions in **b** are truncated to aid visualization (Color figure online)

Substituting fitted growth rates and carrying capacities into the optimal escapement formulas (3) and (4) we calculated optimal escapement in two ways (1) as a proportion of carrying capacity and (2) as a proportion of the maximum population size. For the Bayesian approach, we used the median optimal escapement across the posterior for each dataset. Using this method, we found the Hockey-Stick model fits led to the most conservative (highest) escapement strategies, as a percentage of carrying capacity in 87% of the datasets while Ricker and Beverton-Holt recommended the highest escapement rule in 13% and 0% of the datasets, respectively. Furthermore, in $77\%$ of these fits, Hockey-Stick optimal escapement exceeded 60 percent of carrying capacity (B60, a commonly used sustainability target in fisheries, Fig. 8). Similarly, in the likelihood-based approach, we found that the Hockey-stick model fits suggested the most conservative optimal escapement 90% of the time, Ricker 10% of the time and Beverton-Holt 0% of the time.

Note the optimal escapement distributions for the Hockey-stick model in Fig. 8 are more disperse than for the Ricker and Beverton-Holt models. This is because optimal escapement is a function of the fitted parameters. For Hockey-stick, optimal escapement is the reciprocal of the population growth rate times carrying capacity, as seen in Eq. (4). This means that for slow-growing species, optimal escapement can be close to 100% of carrying capacity (see the green dashed curve in Fig. 2). While for Beverton-Holt and Ricker models, optimal escapement is still a function of the growth rate, this function is much less sensitive to how quickly the species grows at low densities (see red-dotted and blue-solid curves in Fig. 2 and Eq. (3) for Beverton-Holt). Therefore, even though the posterior distributions on *r* appear similar for all three models (compare histograms in Fig. 7), the distribution of optimal escapement values is only disperse for the Hockey-Stick model, more concentrated for Beverton-Holt, and most concentrated for Ricker. This perfectly agrees with the sensitivity of optimal escapement to *r* for each of the three models.

**Fig. 9:**
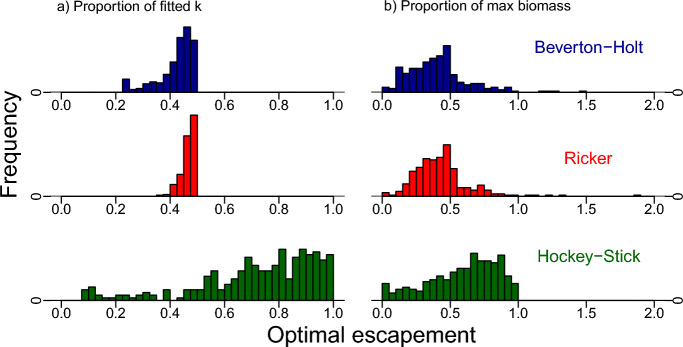
Histogram of optimal escapement for each model calculated using the maximum likelihood estimate of the parameters for each data set **a** as a proportion of the fitted carrying capacity and **b** as a proportion of the maximum observed population size for the data sets in the RAM Stock Assessment Database (color figure online)

Another striking feature of Fig. 8 is the unusual bimodal shape of the distribution for optimal escapement for the Hockey-Stick model. This distribution is an aggregate over all 284 posterior distributions for *r* across the 284 datasets. These posterior distributions incorporate wide, harshly capped, uniform priors on *r*, which preclude any estimates of *r* above or below the bounds of its uniform prior. The small peak of low escapements is caused by both fast-growing species and the fact that the uniform prior gives high weight to large growth rates. High escapements are driven by both slow-growing species and instances of the Markov chain for *r* close to the minimum of the prior. In Fig. 9, we can see the distributions of optimal escapement for the most likely parameter for each species under the likelihood approach. These distributions are no longer as bimodal as in the Bayesian approach. Hence, their shape is purely driven by the frequency of slow and fast-growing species rather than arbitrary reference Bayesian priors.

**Fig. 10:**
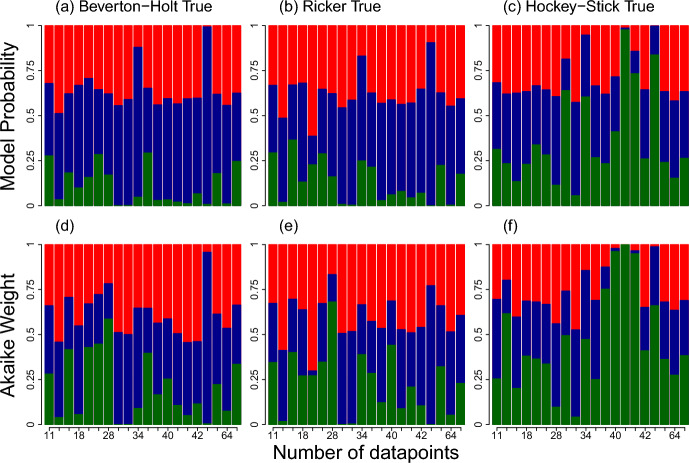
Model probabilities and Akaike weights in the 20 simulated data sets when the data was generated using the **a**, **d** Beverton-Holt, **b**, **e** Ricker and **c**, **f** Hockey-Stick models. Blue (middle), red (top), and green (bottom) bars are for Beverton-Holt, Ricker, and Hockey-Stick Akaike weight and model probabilities, respectively (Color figure online)

Despite the lower estimate of *r* leading to more conservative escapement, the Hockey-Stick’s lower estimate for carrying capacity for many datasets (Fig. 7) counteracts this effect, when escapement is measured in total biomass (rather than the proportion of carrying capacity). We find that when we consider optimal escapement as a proportion of the maximum recorded biomass (i.e. proportional to total escapement), the fitted Hockey-Stick models still lead to more conservative escapements (Fig. 8). In the Bayesian (likelihood) approach Hockey-stick recommended the most conservative escapements in $81\%$ (69%) of the data sets, while Ricker and Beverton-Holt recommended higher escapement rules as a proportion of the maximum observed biomass in 14% (19%) and 8% (12%) of the datasets, respectively. It should be noted that if the true carrying capacity is higher than the maximum observed biomass, the likelihood for the Hockey-Stick model is the same for all values of *k* bigger than the maximum observed biomass (as there would be no density dependence in the observations). Therefore, we capped *k* at the maximum observed biomass when computing its maximum likelihood estimate. For the Bayesian approach, in such datasets, the estimates of optimal escapement, depend on the Bayesian prior, which is uniform over a large support set.

### Simulation Results

When simulating data from the Beverton-Holt model using parameters generated from 20 of the sampled datasets, we found that Beverton-Holt had the highest model probability in 19 of the 20 datasets, and highest Akaike weight in seven (35%) (Fig. 10). While there was considerable support for other models in eight of the 20 runs, there was substantially more support for the true model when Beverton Holt was true, compared to when Ricker or Hockey-Stick was the true model. When Ricker was the true model, it had the highest model probability in only two (10%) of the simulated data sets, but highest Akaike weight in ten (50%). When the Hockey-Stick model generated the data, it only had the highest model probability in seven (35%) of the data sets, but highest Akaike weight in 14 (70%). In both cases, even though Beverton-Holt was not the true model, it still had the highest model probability in 65% and 85% of the simulated data sets, when Hockey-Stick and Ricker models generated the data, respectively.

## Discussion

Classic and widely used population dynamic models lead to harvest recommendations that maintain a population at half of carrying capacity or lower. We showed that the less common Hockey-Stick model often produces more conservative harvest policies that maintain the population size at 1/*r*. This escapement policy leads to higher catches in the case where the model is wrong. It also maintains a stock population size closer to carrying capacity for slow-growing species. Considering that slow-growing species are also often species of conservation concern, this raises the alarm that current management decisions may over-exploit slow-growing populations.

Much of the world’s fisheries have a sustainability target of maintaining a population size of 60 percent of carrying capacity (B60) (Hutton et al. 2019; Streipert et al. 2019), or equivalently 120 percent of the equilibrium population size required to achieve maximum sustainable yield (Kemp et al. 2020). This is meant to be conservative, given that both the Ricker and Beverton-Holt models recommend optimal escapement targets below 50 percent. However, the Hockey-Stick model recommends an optimal escapement higher than 60 percent of carrying capacity when growth rates are below 1.67. Fitted Hockey-Stick growth rates were below 1.67 in 77 percent of the 284 analyzed data sets. This suggests that the sustainability targets of the majority of the world’s fishery management authorities may not be conservative enough to maintain the highest sustainable catches in cases where the Hockey-Stick model is supported.

While Beverton-Holt was the more commonly supported model out of the 284 stock assessment data sets in our analysis, the magnitude of support for Beverton-Holt was only substantially greater than for the Hockey-Stick and Ricker models less than 5% of the time (i.e. a model probability or Akaike weight over 95%). Further, when fitting all three models, using Bayesian inference, to data simulated from the Hockey-Stick and Ricker models, the Beverton-Holt model (the incorrect model) frequently erroneously achieved the highest model probability. In contrast, the likelihood-based approach produced Akaike weights, which more frequently identified the true model that was actually used to simulate the data.

The inability of the Bayesian methodology to identify the correct model in our simulation experiment was likely due to uniform priors on the parameter values biasing model selection towards Beverton-Holt. While the ability of priors to drastically affect model selection is a well-known drawback of using Bayesian inference to perform model selection under limited prior knowledge of parameter values Kass and Raftery (1995), as far as we are aware, this drawback is not widely known in fisheries (Doll and Jacquemin 2019). Our study, therefore, identifies an essential line of open research in Bayesian statistics: developing priors that do not bias model selection in fisheries science and management.

Under the likelihood approach, which is immune to the issue of priors biasing model selection, the Hockey-Stick model best fit 24% of the datasets (compared to 66% and 10% for the Beverton-Holt and Ricker models). Similar to the results under the Bayesian approach, all models typically had considerable support, regardless of which model fit best. The statistical analysis and accompanying simulation experiments suggest that fishery biomass and catch time-series data sets are likely too small and noisy to reliably differentiate the functional form of density dependence in population dynamic models. Under such inconclusive results, managers may want to favour more conservative targets.

Our study was limited to fitting simple, stationary, one-dimensional difference equation models with only two equilibria describing a population growing towards carrying capacity in the absence of harvest. When fisheries are better described by more complicated dynamics such as, multi-species interactions (Holden et al. 2024), ecologically or economically induced Allee effects (Perälä et al. 2022; Holden and McDonald-Madden 2017), non-stationary parameters (e.g. due to time-dependent environmental regime shifts, Johnson et al. 2015), time delays (e.g. due to age-structure,Tahvonen 2008; Filar et al. 2024), or alternative stable states (Gårdmark et al. 2015), our analysis would not be appropriate. Such complex dynamics can drive fisheries collapse and impair recovery. Unfortunately, the complicated models capable of describing these processes can be more challenging to implement due to identifiability issues and overfitting (Clark et al. 2010; Koetke et al. 2020). Therefore, in such cases, other management tools, such as marine reserves, may be more effective for preserving desired ecological states (Erm et al. 2023, 2024; Zhao et al. 2024), compared to harvest regulations guided by simple population dynamic models.

Despite the caveats, Ricker and Beverton-Holt models are the most widely used density-dependent population dynamic models in fisheries since the 1950’s (Needle 2001). These models are popular because they are historical, simple, can be derived from mechanistic ecological principles (Anazawa 2012), and are easily fitted to data (Needle 2001). Similar to our results, previous works have challenged classic density-dependent population dynamic models’ fit to real-world time-series data across species (Sakuramoto 2005; Carruthers et al. 2014; Knape and de Valpine 2012), and have also shown that the Hockey-Stick model fits recruitment data similarly well compared with the more classic models (Barrowman and Myers 2000). But past works have focussed on the fact that Ricker and Beverton-Holt models predict higher growth rates (Barrowman and Myers 2000) than the Hockey-Stick model, and therefore can overestimate resilience and underestimate extinction risk for exploited populations (Clark et al. 1973). Additional work has also shown that the harvest rules derived from these models can be too aggressive when accounting for long-run uncertainties in observed biomass (Memarzadeh and Boettiger 2019). Our work provides yet another reason why such harvest policies may be too aggressive. Even when ignoring the risk of stock collapse, measurement uncertainties, and fluctuations, classic sustainability targets can still reduce long-term catch compared to more conservative policies if density dependence acts on populations according to a non-smooth function, such as the Hockey-Stick model.

## Data Availability

All data files and code used to produce this manuscript can be found here https://github.com/v-cattoni/Revisiting-sustainability-targets-for-harvested-populations.

## Appendix: Optimal Harvest in the Hockey-Stick Model

Consider the model in Eq. (1). To solve for the harvest at each time step, $h_t$, $0 \le h_t \le x_t$, that maximizes the total discounted catch, over a fixed time horizon *T*,

$$\begin{aligned} \mathbb {E}\left\{ \sum \limits _{t=0}^{T} \rho ^t h_t \right\} , \end{aligned}$$

where $\rho$ is the discount factor, it is useful to define escapement as,

$$s_t = x_t - h_t.$$

This problem is equivalent to solving for the optimal escapement, $s_t$, at each time step *t*, since each escapement uniquely determines a harvest and vice versa. In order to solve this problem, we use stochastic dynamic programming. Define a value function,

$$\begin{aligned} V(x_T) = \max _{s_T}\{x_T-s_T\}, \end{aligned}$$

and, for all $t\le T$,

$$\begin{aligned} V(x_t) = \max _{s_t} \{ x_t - s_t + \rho \mathbb {E}\left[ V(x_{t+1})\right] \}. \end{aligned}$$

Clearly at time *T* it is optimal to harvest everything, meaning $V(x_T)=x_T$ and $s_T=0$. Therefore, at time $T-1$ we have

$$\begin{aligned} V(x_{T-1})&= \max _{s_{T-1}}\{x_{T-1}-s_{T-1}+\rho \mathbb {E}\left[ V(x_{T})\right] \}\\&= \max _{s_{T-1}}\{x_{T-1}-s_{T-1}+\rho \min \{rs_{T-1}, k\}\}\\&= \max _{s_{T-1}} \left\{ \begin{array}{lr} x_{T-1} + (\rho r - 1)s_{T-1}, & \text {if }\, s_{T-1} < k/r\\ x_{T-1}-s_{T-1}+\rho k, & \text {if }\, s_{T-1} \ge k/r \end{array} \right\} \end{aligned}$$

Noting that $x_{T-1}$ is a known constant, it is optimal to increase escapement if $s_{T-1}<k/r$ and decrease escapement if $s_{T-1}>k/r$. Therefore, $s_{T-1}=k/r$. Applying the same logic, backward in time, yields the same optimal escapement, $s_{t}=k/r$ for all $t<T$. Note that in the above argument we made the simplifying assumption that the population is self-sustaining, $Z_t \ge 1/r$ for all *t*, as is typical for these types of arguments (Reed 1979). While this is quite a strong assumption, it turns out that it can be verified numerically that the optimal policies are near-optimal even when the assumption is violated (Holden and Conrad 2015).

### Data Fitting Details

This section describes the specific implementation used for Bayesian inference with the Rstan and bridgesampling packages. The Rstan package uses the no-u-turn sampler (Hoffman et al. 2014) so it samples from a Markov chain that is designed to have the posterior distribution as its limiting distribution. We use four chains, each with 100,000 iterations and a burn-in of 50,000 iterations. We select max_treedepth=30 and adapt_delta=0.999 for robustness, and thin=10 to reduce storage requirements. We use the default options provided by the Stan team for all other function arguments. We use all defaults for the bridgesampling package to calculate the logarithm of the normalising constant for each model and dataset combination.

To assess the convergence of the set of Markov chains for each dataset and model combination, we calculated the $\hat{R}$ diagnostic (Gelman and Rubin 1992) for each of the three parameters of interest and the log (unnormalised) probability. Across the 284 observed datasets, the number of runs with at least one $\hat{R}>1.01$ was zero for the Ricker model, one for the Beverton-Holt model and eight for the Hockey-Stick model. Only one run for the Beverton-Holt model and one run for the Hockey-Stick model failed to meet the less stringent threshold of $\hat{R}<1.1$ recommended by Gelman and Rubin (1992), with a maximum of $\hat{R}\approx 1.2$ observed. This indicates reasonable convergence for the large majority of the $284 \times 3 = 852$ sets of Markov chains.
